# Cox-2 inhibitors in mandibular third molar surgery

**DOI:** 10.25122/jml-2019-0007

**Published:** 2019

**Authors:** K. Janarthanan, S. Adalarasan

**Affiliations:** 1. Reader, Department of OMFS, RMDC & H, Annamalai University, India; 2. Lecturer, Department of OMFS, RMDC & H, Annamalai University, India

## Abstract

Pain control during and after any surgical procedure, is extremely essential for the comfort of patients. Pain killers used routinely act by inhibiting cyclooxygenase to control pain and inflammation. Cox-1 is constitutively expressed in most cell types, including platelets, whereas Cox-2 is absent from most healthy tissues but is induced by pro-inflammatory or proliferative stimuli. Cox-1 plays a role in the production of prostaglandins involved in protection of the gastric mucosal layer and thromboxanes (TX) in platelets. Cox-2 generally mediates elevations of prostaglandins associated with inflammation, pain, and pyresis. Nonsteroidal anti-inflammatory drugs (NSAIDs) such as aspirin and ibuprofen are generally nonselective inhibitors of Coxs. This lack of selectivity has been linked to their propensity to cause gastrointestinal side effects. The new Cox-2 selective inhibitors, or coxibs, show the same anti-inflammatory, analgesic, and antipyretic effects as nonselective NSAIDs but are supposed to have reduced side-effect profiles. This study evaluates whether rofecoxib (50 mg) given one hour pre-operatively or the same drug given one hour post-operatively is more effective in controlling the pain and swelling in mandibular third molar surgery.

## Introduction

Excellent pain control in dentistry is essential for the delivery of optimal dental care and for the well being of the patient. It is now known that high levels of cyclooxygenase -2 (Cox–2) are expressed at the sites of tissue inflammation leading to the synthesis of prostaglandins that mediate pain and inflammation.

Prostaglandin synthesis in humans is catalyzed by two distinct isoforms of cyclooxygenase (Cox-1 and Cox-2). Cox-1 is constitutively active throughout the body, whereas Cox-2 expression is markedly upregulated by a variety of inflammatory mediators. These distinct expression patterns have led to the proposal that prostaglandins produced by Cox-1 are largely responsible for physiological functions, while Cox-2 derived prostaglandin mediate pathophysiological and inflammatory processes including pain.

Cox-2 inhibitors like rofecoxib act by specifically blocking Cox-2 activities without blocking the other isoform of cyclo-oxygenase, namely Cox-1, which is responsible for gastric mucosal protection and vascular homeostasis. So we did a comparative study on the efficacy of rofecoxib, a Cox-2 inhibitor by administering the drug preoperatively for one group of patients and postoperatively for the other group.

## Methodology

The study group comprised of those patients who came to the department of oral and maxillofacial surgery, Mahatma Gandhi Dental College and Hospital, Pondicherry for the surgical removal of impacted mandibular third molar. A total of 38 patients, that is, 19 patients in each group were included in the study. A randomization table was prepared manually and a randomly allocated number was given to each patient using this table. A proper clinical and radiologic evaluation was done preoperatively. The patients underwent the treatment under local anesthesia (2% lignocaine containing 1:80,000 adrenaline).

### Inclusion Criteria

The inclusion criteria were as follows:

1. All types of impaction were taken up for the study.
2. Patients of age 18 – 35 were included.
3. Both sexes were included.
4. Only patients willing to give an informed consent were made to participate in the study.

### Exclusion Criteria

The exclusion criteria were as follows:

1. Hypersensitivity to analgesics.
2. Patients who have taken tricyclic antidepressants, sedatives, analgesics, antibiotics, antihistamines, or corticosteroids in the last one month.
3. Medically compromised patients.
4. Pregnant ladies

The two groups in the study were:

#### Group – I

Rofecoxib 50 mg orally, once daily and it was given 1 hour pre-operatively. A placebo was given 5 minutes after the surgery.

#### Group – II

For the patients in this group, the placebo was given one hour preoperatively. rofecoxib 50 mg orally, once daily was given five minutes post operatively.

The patients in both the groups continued taking rofecoxib 50 mg (once daily) for another five days.

In this triple blind study, the drugs were given to the patients by a post graduate teacher/staff nurse in the department who was neither the operator nor the observer.

### Preoperative Details

The following details were recorded preoperatively:

Name of the patient, age, sex, address, date, randomization number, informed consent, tooth number, operator’s name and experience, type of impaction, and split/elevated.

### Surgical Procedure

After giving inferior alveolar nerve block using 2% lignocaine hydrochloride containing 1:80,000 adrenalines, ward’s incision was placed in relation to the impacted third molar. The flap was raised and the tooth was removed either by splitting or elevation after bone removal. The wound was closed with 3-0 black braided suture.

The surgical procedure was done by post graduates in the department. The operators were requested not to prescribe any pain killer or other drugs after the surgery. The operators were unaware of which drug was given preoperatively and post operatively.

The patients were asked to assess their pain intensity at 5, 7, and 24 hours after surgery. The subjects were asked to record their pain Intensity (PI) on a 100 mm visual analog scale (VAS) by placing a mark on the line to indicate the magnitude of their pain.

**Figure:**
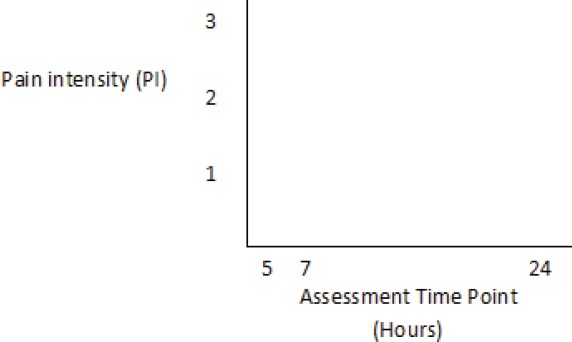


The subjects were asked to assess their pain intensity on a four-point categorical scale in the graph drawn above. The scoring scale was as follows:

Score 0 – No pain.

Score 1 – Mild pain.

Score 2 – Moderate pain.

Score 3 – Severe Pain.

The patients were recalled 48 hours after the administration of the drug and they were asked about the pain relieving effect of the drug by a single observer who was unaware of the drug given. The graph was also seen by the observer.

However, patients were advised to take a rescue medication (one Paracetamol tablet) if pain developed before the time at which rofecoxib was supposed to be taken. If the patient had taken the rescue medication, the pain intensity (PI) just before taking the rescue medication had to be marked on the graph by the patient himself/herself.

### Postoperative Details

The following details were recorded postoperatively:

1. Duration taken for surgery.
2. Pain intensity, after 5, 7, and 24 hours as marked in the visual analog scale.
3. Whether patient has taken rescue medication.
4. CRP test was done after 48 hours to assess the inflammatory response.

C-reactive protein is one of the family of acute phase proteins found in the blood of both humans and animals. It is found in concentrations of less than or equal to 5 µg/ml in the serum of healthy persons. However, during an inflammation or infection, the levels may increase by as much as one thousand fold and can be detected as early as 5–10 hours after tissue damage. The increase in CRP levels in serum appears to be a non specific phenomenon but the change can be used to monitor the course of certain diseases and their treatment.

Elevated level of CRP can be demonstrated in cases of acute myocardial infarction, rheumatoid arthritis, bacterial and viral infections, acute rheumatic fever, and several types of malignancies.

In our study, we have assessed the CRP level of the patients both qualitatively and semi quantitatively using the ‘SPAN’ CRP test kit.

## Results

Analgesics are routinely used after dental and oral surgical procedures. But the NSAIDs which are usually prescribed, though effective in controlling inflammation, have some undesirable side effects. So, we decided to evaluate the efficacy of coxibs (rofecoxib 50 mg) in controlling the inflammation after removal of impacted mandibular third molar by comparing two groups – one receiving the tablet 1 hour preoperatively and the other receiving the tablet 5 minutes postoperatively. The patients in both groups continued taking the tablet for a total period of 5 days.

The mean age of patients in our study was 26.5 years, ranging from 18 years to 35 years. The number of males and females were 15 and 23, respectively, with a ratio of 1:1.5. For 31 patients the tooth was removed by splitting and for the other seven patients the tooth was removed by elevation. The type of impaction was mesioangular in 27 cases, distoangular in 10 cases, and horizontal in one case.

The patients reported for follow-up after 48 hours and then after 1 week following surgery. One patient had numbness in the lower lip on the operated side and six patients had postoperative infection. These complications were managed conservatively.

The maximum pain score as assessed using the visual analog scale was ‘1’ in the postoperative group and ‘2’ in the preoperative group, twenty four hours after surgery. The C-reactive protein level 48 hours after surgery ranged from <6µg/ml-96 µg/ml in the postoperative group and <6 µg/ml-48 µg/ml in the preoperative group.

Two patients in the postoperative group and four patients in the preoperative group had taken rescue medication once after surgery. One patient in each of the group had taken rescue medication twice after surgery.

A comparison was done between the two groups based on CRP level and the number of patients who required rescue medication. There was no statistically significant difference between both the groups in the level of inflammation after surgery (p <0.05 is statistically significant).

**Table:** Rofecoxib Preoperative Group

Name of the Patient	Age/Sex	Pain Level (After 24 Hrs)	Rescue Medication	Post OP CRP Level
R.SS Verma	23/M	0	Nil	12 μg/ml
Lilly	25/F	1	2	48 μg/ml
Maxim Gandhi	25/M	0	Nil	24 μg/ml
Jayalakshmi	26/F	2	Nil	< 6 μg/ml
Natraj	33/M	0	Nil	24 μg/ml
Rani	35/F	1	Nil	12 μg/ml
Kamala	27/F	1	Nil	12 μg/ml
Bhuvaneswari	26/F	2	Nil	6 μg/ml
Ganesh	26/M	0	1	12 μg/ml
Reena	20/F	0	1	12 μg/ml
Devi	24/F	0	Nil	12 μg/ml
Sekar	28/M	0	Nil	< 6 μg/ml
Abdul Kareem	32/M	1	1	48 μg/ml
Sesiline	21/F	0	Nil	24 μg/ml
Tamilarassi	20/F	2	Nil	48 μg/ml
Murugan	30/M	1	Nil	24 μg/ml
Kala	23/F	1	Nil	24 μg/ml
Suresh	26/M	0	1	48 μg/ml
Murali	19/M	0	Nil	12 μg/ml

**Table:** Rofecoxib Postoperative Group

Name of the Patient	Age/Sex	Pain Level (After 24 Hrs)	Rescue Medication	Post OP CRP Level
Balakumar	27/M	0	Nil	24 μg/ml
Laxmi	26/F	1	Nil	24 μg/ml
Devi	28/F	0	Nil	6 μg/ml
Saraswathi	23/F	0	Nil	< 6 μg/ml
Jayalakshmi	32/F	0	Nil	6 μg/ml
Vasugi	23/F	1	Nil	12 μg/ml
Sumathi	20/F	0	Nil	12 μg/ml
Mathiselvi	26/F	0	Nil	24 μg/ml
Varalakshmi	24/F	1	Nil	12 μg/ml
Mohammed Malik	27/M	0	Nil	6 μg/ml
Devikala	23/F	1	Nil	6 μg/ml
Suganthiya	18/F	1	Nil	< 6 μg/ml
Sarbunisha	33/F	0	2	12 μg/ml
Priya	24/F	0	Nil	12 μg/ml
Sakthimurugan	27/M	1	Nil	24 μg/ml
Moorthy	33/F	1	Nil	96 μg/ml
Raji	24/F	1	1	6 μg/ml
Manikandan	20/M	1	Nil	< 6 μg/ml
Geetha Lakhsmi	26/F	0	1	48 μg/ml

### Statistical Analysis

Development of the coxibs, a new group of anti-inflammatory drugs, represents a response to the unsatisfactory therapeutic profile of NSAIDs. Although NSAIDs are the most commonly used analgesic agents in ambulatory patients, their long term use is limited by gastrointestinal effects such as dyspepsia and abdominal pain and, less often, gastric or duodenal perforation or bleeding.

**Table 1:** CRP – Level

CRP Level	Rofecoxib PreOperative	Rofecoxib Postoperative	Total	Chi-Square Value
Less than 6 μg/ml	2 (10.5)	3 (15.8)	5 (13.2)	0.9037 (p=0.64)
6–24 µg/ml	13 (68.4)	14 (73.7)	27 (71.0)	
48-96 µg/ml	4 (21.0)	2 (10.5)	6 (15.8)	
Total	19 (100.0)	19 (100.0)	38 (100.0)	

Chi-square value is insignificant.

Chi-square table value for degrees of freedom 2 @ 5% level is 5.99

Conclusion:- Giving rofecoxib to the patients 1 hour before and 5 minutes after the surgery does not have significant influence (change) in CRP level. That is, CRP level and timing of medication are independent of each other. There is no relationship.

**Table 2:** Relationship between timing of rofecoxib and rescue medication.

Rescue Medication	Rofecoxib Preoperative	Rofecoxib Postoperative	Total	Chi-Square Value
Yes	5 (26.3)	3 (15.8)	8 (21.1)	0.6333 (p=0.42)
No	14 (73.7)	16 (84.2)	30 (78.9)	
Total	19 (100.0)	19 (100.0)	38 (100.0)	

Chi-square is insignificant.

Chi-square table value for degree of freedom 1 @ 5% level is 3.84

Conclusion:- The Rescue medication and timing of rofecoxib are independent.

In the recent past, the analgesic efficacy of coxibs had been compared with NSAIDs in the dental pain model. Malmstrom K., et al., compared the analgesic efficacy of rofecoxib with celecoxib and ibuprofen in patients experiencing pain after third molar surgery. They found that rofecoxib had superior analgesic effect and longer duration of action than celecoxib and ibuprofen, respectively.

In our study we had assessed the efficacy of the cox-2 inhibitor rofecoxib based on the timing of its administration unlike most of the studies which had either compared its efficacy with another cox-2 inhibitor or a NSAID.

Swan et al., by comparing the effects on renal function in patients receiving rofecoxib, indomethacin, and placebo concluded that rofecoxib has effects on renal function quite similar to nonselective NSAIDs

The ‘CLASS’ trial which lasted for 13 months concluded that there was no statistically significant difference between celecoxib and NSAIDs like ibuprofen and diclofenac in the incidence of upper gastrointestinal bleeding or gastric perforation.

However, Claire Bombardier established a statistically significant difference in the upper gastrointestinal events by comparing rofecoxib with naproxen in patients with rheumatoid arthritis.

Francesca Catella – Lawson had demonstrated that the concomitant administration of rofecoxib did not affect the pharmacodynamics of aspirin in patients with both arthritis and vascular diseases. However, we have excluded medically compromised patients from our study group.

Muhammad. Mamdani did an observational cohort study over a period of 1 year to compare the rates of upper gastrointestinal hemorrhage in patients treated for arthritis with either cox-2 inhibitors or NSAIDs and concluded that there is a lower short term risk of upper gastrointestinal hemorrhage for selective cox-2 inhibitors compared with non-selective NSAIDs.

Apart from control of inflammation and pain associated with it, the antiangiogenic activity of cox-2 inhibitors provides an additional rationale for the use of cox-2 inhibitors in severe painful conditions like rheumatoid arthritis. James. M. Woods demonstrated that rofecoxib acts directly on human dermal microvascular endothelial cells and inhibit their chemotactic and tube forming ability.

The cox-2 inhibitors are believed to be associated with an increased risk of myocardial infarction and congestive cardiac failure. [29,34] Hyon K. Choi (2004) [15] showed that naproxen is associated with a longer life expectancy than rofecoxib in patients with arthritis except in those at a low risk of myocardial infarction.

Muhammad Mamdani (2004) [29] suggested a higher risk of admission for congestive cardiac failure in users of rofecoxib. But further studies with long term follow up are required to establish the relationship between coxibs and cardiac conditions like myocardial infarction and congestive heart failure.

Gizzarelli compared the efficacy of preoperative administration of rofecoxib with ibuprofen in third molar extraction. The results showed that at 6 hours rofecoxib group had significantly better pain relief than ibuprofen group.

Kyle.S. Christensen in a randomized placebo-controlled trial on patients experiencing moderate or severe pain within 4 hours after multiple third molar extractions showed that valdecoxib had a significantly faster onset of action and superior analgesic effect than rofecoxib.

There are studies ‘for’ and ‘against’ the use of cox-2 inhibitors as an alternative to NSAIDs in patients who develop adverse drug reaction to NSAIDs. However, in our study we had not been confronted with a situation in which an allergic reaction to rofecoxib occurred.

## Conclusion

This study group comprised of those patients who came to the department of oral and maxillofacial surgery, Mahatma Gandhi Dental College and Hospital, Pondicherry for the surgical removal of impacted mandibular third molar. The study group comprised of one group for which rofecoxib 50 mg was given one hour preoperatively and the other for which the same drug was given 5 minutes postoperatively.

Five patients in the preoperative group and three patients in the postoperative group required rescue medication despite taking rofecoxib 50 mg once daily for five days including the day of surgery. No significant statistical difference was observed in the CRP level between both the groups.

Our study concluded that the timing of administration of drug (preoperatively or postoperatively) does not have any significant difference in controlling the pain and swelling in mandibular third molar surgery.

## Conflict of Interest

The authors confirm that there are no conflicts of interest.
